# Long COVID treated successfully with antivirals in a rituximab-treated follicular lymphoma patient with persistent negative-antibodies to SARS-CoV2

**DOI:** 10.1016/j.heliyon.2023.e17149

**Published:** 2023-06-21

**Authors:** Elias Tayar, Ryan Isber, Nidal Isber

**Affiliations:** a Hamad Medical Corporation, Doha, Qatar; b Binghamton University, New York, USA; c Richmond University Medical Center, New York, USA

**Keywords:** COVID-19, Antivirals, Follicular lymphoma, Long COVID

## Abstract

Long COVID is a well-known complication to COVID-19 that affect millions of people worldwide and causes wide range of symptoms.

We present a rare case of a previously diagnosed follicular lymphoma patient, who had a long COVID with persistent negative SARS-CoV-2 antibodies and required an aggressive antiviral treatment.

## Highlights

• Persistence of SARS-CoV-2 infection among immunocompromised patients are due to inability of the immune system to eliminate the virus.
• Treatment with anti-CD20 monoclonal antibodies (as in lymphoma patients) causes lack in antibody response to both the COVID-19 vaccine and acute COVID-19 infection.
• Aggressive treatment with antivirals and long-acting monoclonal antibodies against SARS-CoV-2 may be lifesaving among these vulnerable immunocompromised patients.

## Abbreviations

COVID-19: Corona virus disease 2019
SARS-CoV2: severe acute respiratory syndrome coronavirus-2
PASC: post-acute sequelae of COVID-19
RT-PCR: Reverse Transcriptase- Polymerase Chain Reaction

## 1 Introduction

COVID-19 due to novel severe acute respiratory syndrome coronavirus-2 (SARS-CoV-2) has been circulating the world for more than three years now, and has been responsible for millions of deaths worldwide [1], In the majority of cases of COVID-19, symptoms subside within days to weeks; but in some cases symptoms can persist for months after the resolution of the acute infection. These patients with prolonged symptoms are said to have post-acute sequelae of COVID-19 (PASC) or long COVID which involves a complex heterogenous symptoms that can be disabling [4]. The symptoms often include brain fog, fatigue, headaches, dizziness, shortness of breath, depression, anxiety, sleep problems and autonomic dysfunctions among many others [2,3].

Recent estimates shows that around 16 million working-age Americans (those aged 18 to 65) have long COVID today. Of those, 2 to 4 million are out of work due to long COVID [4].

Currently, the underlying biological mechanism of long COVID is unclear, however multiple hypotheses have been proposed for its pathogenesis, including persistent virus or virus remnants, autoimmunity, dysbiosis, latent viral reactivation and unrepaired tissue damage [[5], [6], [7], [8], [9], [10], [11], [12]].

Some researchers speculate that symptoms of long COVID may arise from the immune system's ongoing reaction to the virus that remains in the body after the acute phase of the infection is over [13].

We present a case of long COVID in a patient with Rituximab-treated follicular lymphoma who was fully vaccinated but remains negative for SARS-CoV-2 antibodies. Treatment with a combination of antiviral medications plus long-acting monoclonal antibodies against SARS-CoV-2 resulted in complete and sustained resolution of long COVID symptoms.

Our case supports the hypothesis that persistence of SARS-CoV-2 infection, due to the inability to eliminate the virus, may be the biological mechanism of some cases of long COVID especially in immunocompromised patients who may lack antibody response to both the COVID-19 vaccine and acute COVID-19 infection such as the case of our patient.

## 2 Case presentation

This is a case of a 53-year-old female who is known to have low-grade stage 3 follicular lymphoma since 2017. She was on “watch and wait” protocol until January 2020 when her lymphoma was noticed to be progressing. At that time, she was started on chemo-immunotherapy treatment with Bendamustine/Rituximab. After achieving complete therapeutic response, the patient was continued on Rituximab maintenance therapy every 8 weeks for a period of 2 years. Her last Rituximab dose was in January 2022. The patient received two doses of viral vector vaccine (AstraZeneca), her second dose of vaccine was in June 2021.

On March 10th, 2022, Patient presented to the emergency department complaining of low-grade fever, mild cough, and fatigue for a period of 3 days. Her body temperature, blood pressure, heart rate, and peripheral oxygen saturation were 38.2 °C, 122/78 mm Hg, 86 bpm, and 96% on room air, respectively. Auscultation of the lungs and the heart showed clear lung fields and no abnormal cardiac murmurs respectively.

**Fig. 1:**
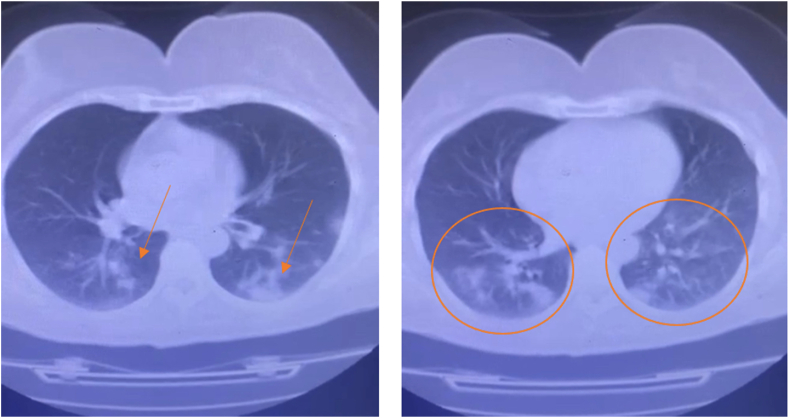
Chest CT (2 slides) (March 10th,2022).

Laboratory tests showed mildly elevated CRP (14 mg/L), white blood cell count of (6.2 × 10^^^9/L) with absolute lymphocyte count of (1.42 × 10^^^9/L) (23% of total white blood cells) (Table 1). Chest computed tomography revealed ground glass opacities and ‘crazy paving’ pattern bilaterally in the lower lobes (Fig. 1).

**Table 1:** Patient laboratory tests.

Date (2022)	10 Mar	20 Mar	2 Apr	8 Apr	18 Apr	28 Apr	3 May
Lab tests							
Hemoglobin (g/dl)	12.6	12.7	12.1	11.3	11.5	11.5	11.6
WBCs (10^^^9/L)	6.2	6.7	12.6	13	8.3	8	7.5
Neutrophils (%)	73	78	88	89	83	78	75
Lymphocytes (%)	23	20	7	6	10	18	21
CRP (mg/L)	14	14.3	60	98	40	20	5
D-Dimer (ng/ml)	450	680	800	650	400	270	230
COVID-19 RT PCR	+	+	+	+	+	–	–
CT- values	25.34	24.5	16.55	22.5	27.7	–	–
COVID-19 Antibody test	–	–	–	–	–	–	+

The result of the reverse transcription-polymerase chain reaction (RT-PCR) test of the sputum was positive for SARS-CoV-2.

Patient received symptomatic treatment with antipyretics and was sent home for self-isolation. Her symptoms were considered mild, so she was considered by the treating team not to be eligible to receive antivirals according to the national guidelines and due to shortage of supplies. She was asked to follow-up after 10 days or in case of symptoms deterioration. However, her mild symptoms persisted, and her RT-PCR remained positive for weeks.

On April 2nd, Patient developed high-grade fever (39.6 °C) and her inflammatory markers trended up (Table 1). Chest-x-ray showed some haziness in both lower lung fields with infiltrations in around 10% of both lungs which goes with atypical viral pneumonia (Fig. 2a). Patient was prescribed Dexamethasone 6mg orally for 10 days along with antipyretics when needed. Few days later, the patient had significant clinical improvement associated with decline in her inflammatory markers.

**Fig. 2A:**
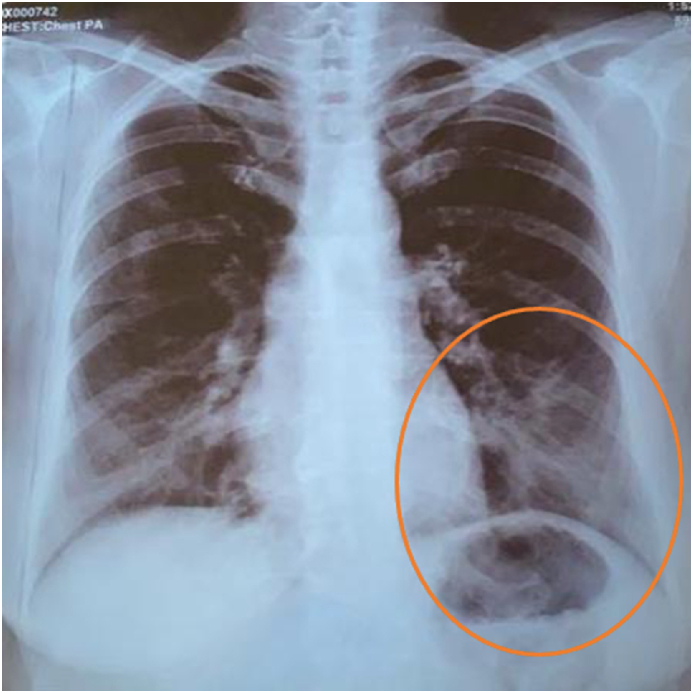
Chest X-Ray (april 2nd,2022).

After the completion of full-course of Dexamethasone, her symptoms of low-grade fever and fatigue returned, and chest x-ray showed bilateral ground-glass appearance with some progression in haziness in both lower and middle lung fields (Fig. 2b); her RT-PCR test remained positive, and her COVID-19 antibody test was still negative after around 40 days of her first positive COVID-19 RT-PCR test. At this time patient had complete work up which excluded all kinds of infectious etiologies and excluded recurrence of lymphoma.

**Fig. 2B:**
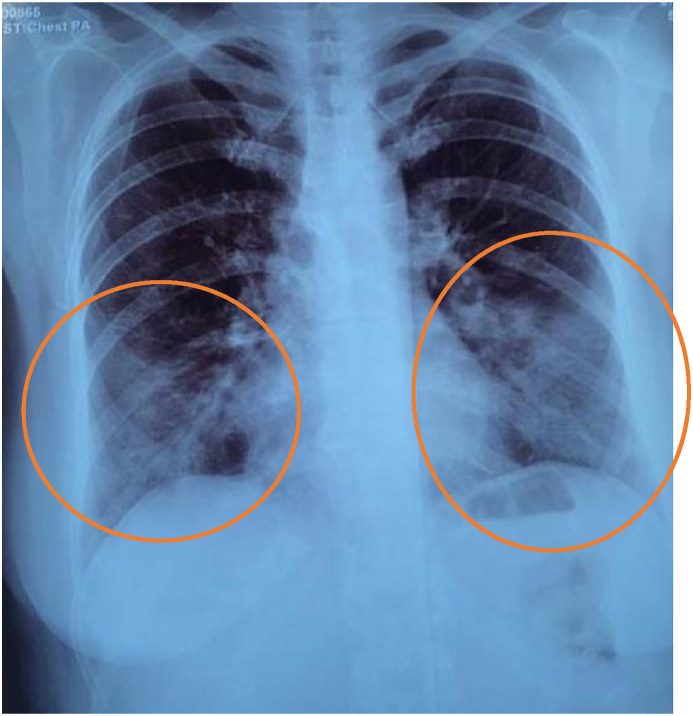
Chest X-ray (april 26th,2022).

The lack of antibody response during her illness was interpreted by the first treating team as the patient was having something else unrelated to COVID -19, or perhaps long COVID. However, the patient was told that there is no specific therapy for long COVID. After experiencing a long period of apprehension and uncertainty about the fate of the patient, the family sought a second opinion. Upon reevaluation by a new consultant, it was hypothesized that the patient may have a persistent SARS-CoV-2 infection. In order to prove or exclude this hypothesis, treatment trial with antiviral medications should be attempted.

The new treating physician who took over the case made the recommendation to treat with several antiviral agents which has different mechanisms of action, this decision was based on the judgement that the patient is immunocompromised, and one antiviral might not be effective alone in eliminating the virus. In addition, there was a concern about a rebound phenomenon that has been reported with treatment with Paxlovid. The patient was initially treated with Remdesivir which was the first available antiviral drug. Remdesivir was administered for 10 days (200mg day 1 then 100 mg for 9 days) which resulted in significant improvement in the patient's symptoms. Her antibodies to SARS-CoV-2 continued to be negative.

Following treatment with Remdesivir, the patient received 5-day course of the Pfizer oral antiviral Paxlovid® (300 mg Nirmatrelvir and 100 mg Ritonavir twice daily), followed by one dose of Evusheld® “300mg Tixagevimab/300mg Cilagavimab” administered as two separate consecutive intramuscular injections.

After administration of the above therapy, her antibody titer against SARS-CoV-2 became positive, due to treatment with Evusheld®, and her COVID-19 RT PCR test became negative. The type of serology test used to detect antibodies evaluates both IgM and IgG responses to two viral proteins Spike and Nucleocapsid, respectively in one single test, and it was the test that was administered during all periods of patient's illness.

Patient responded well to antiviral therapies. Her temperature returned to normal, her fatigue has resolved, and she became active and feeling well similar to baseline prior to her illness. She also had normal physical examination and her new chest x-ray showed an improvement in bilateral infiltrations as in (Fig. 2c). The patient remains completely asymptomatic at 3 and 6 month follow up visits.

**Fig. 2C:**
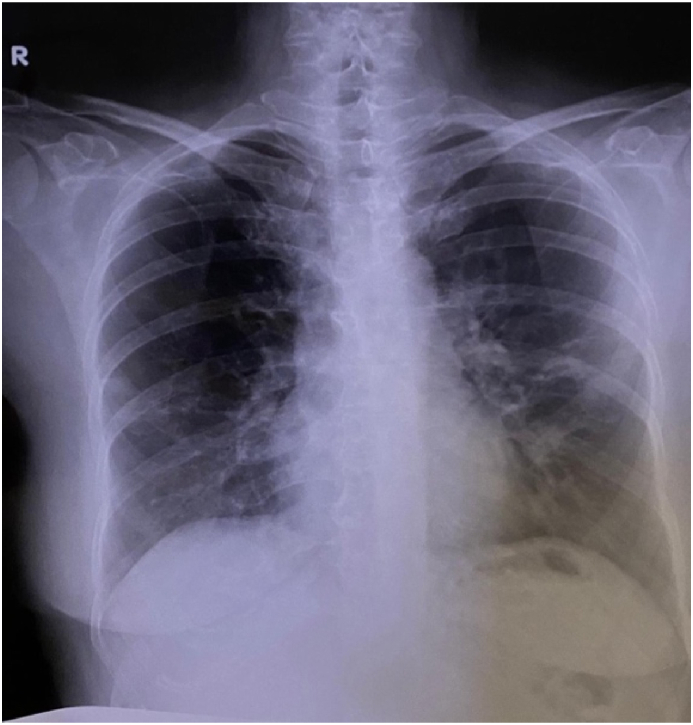
Chest X-ray (may 14th,2022).

In brief, our vaccinated patient who has follicular lymphoma in remission, developed protracted low-grade symptoms later after outpatient treatment of acute COVID-19. Her symptoms persisted long enough by definition to suggest that she had long COVID. Treatment with various antiviral agents against SARS-CoV-2 resulted in complete resolution of her symptoms.

## 3 Discussion

Most patients with acute phase of COVID-19 recover, however subsets of convalescent patients may continue to suffer from protracted symptoms after their initial recovery from the acute illness even among those with initially mild disease. Those patients are said to have long COVID [14].

People with long COVID have persistent symptoms long after the initial acute illness with COVID-19. Symptoms often include brain fog, fatigue, headaches, dizziness, shortness of breath, depression, anxiety and sleep problems [15]. A recent systematic review with meta-analysis showed that more than 60% of patients who survived COVID-19 continue complaining of one post-COVID-19 symptom at least for more than one month after its onset [16]. Currently, there is no specific therapy to treat long COVID [17].

Several studies show that patients with lymphomas and hematologic malignancies in general are at higher risk of mortality when they develop COVID-19 [18]. Hematological malignancies impair the function and production of blood cells that fight infections. These patients tend to have more comorbidities than their matched population and tend to have more hospitalization rates [19]. Lymphoma patients are usually immunocompromised due to the lymphoma biological features such as lymphopenia or due to its treatment regimens, which may lead to more severe and prolonged infections [20].

Chemotherapy, along with Rituximab are the cornerstone in the treatment of most lymphomas. Rituximab is an Anti-CD20 monoclonal antibody that induces a rapid depletion of more than 95% of CD20-positive mature B-cells, altering humoral and cellular response to new pathogens [21], This will make many patients treated for B-cell lymphomas prone to have prolonged infections due to their impaired ability to produce antibodies against pathogens and after vaccination.

According to a study that has examined the antibody response to SARS-CoV-2 vaccines in patients with hematologic malignancies [22], 25% of blood cancer patients particularly in patients with B cell malignancies, who received COVID-19 vaccinations, failed to produce detectable antibodies. Another study from Stanford medicine [23] found that Rituximab blunts or eliminates the antibody response to COVID-19 vaccines, if it is administered up to a year prior to vaccination.

In a recent study by Shree et al. [24] the antibody response to COVID-19 vaccine was measured in 126 lymphoma patients who had been treated with rituximab. The team found that 55% of all the patients developed antibodies that would block the virus's spike protein from binding to its specific receptor. However, none of the patients who received Rituximab six or fewer months before being vaccinated generated any neutralizing antibody response. The time elapsed since the patient's last rituximab treatment was a significant predictor of vaccine response. They found that receiving Rituximab treatment 12 or fewer months before vaccination was associated with low likelihood of mounting an antibody response to the vaccine [24].

It has been speculated that the symptoms in long COVID may arise from the immune system's ongoing reaction to SARS-CoV-2 virus that remains in the body after the acute phase of the infection is over [25]. One study showed persistent shedding of the virus from the gastrointestinal tract for up to seven months in some people. Knowing that, it seems reasonable to expect that treatment with antiviral medication in these cases may benefit patients with long COVID [25].

Although it has been established that Paxlovid® treatment during acute COVID-19 lowers hospitalization rates [26], there is an emerging question whether Paxlovid® therapy for acute COVID-19 lowers the risk of long COVID. There are no data available at present to answer this question. However, it makes sense that Paxlovid® might lower the risk of long COVID by quickly reducing the viral load or decreasing the chance of infection from becoming persistent.

Our case introduces potential evidence to support that. Our patient suffered from protracted symptoms after the treatment of acute COVID, so she had what it seemed a case of long COVID. The successful treatment of her case of long COVID with antiviral agents, would have possibly been successful if the same treatment would have been introduced earlier during the acute phase of the infection.

There are some anecdotes including a case series posted on May 3, 2022 on Research Square, researchers from University of California [27] followed three patients in their 40s who had tested positive for COVID and later struggled with debilitating symptoms, consistent with long COVID. In two of the three patients, Paxlovid was taken weeks after the start of symptoms, contrary to its indications. One patient was prescribed the antiviral after re-exposure to the virus, more than seven weeks after symptom onset, and his health improved until he felt almost back to normal. A second patient took Paxlovid approximately three weeks after symptom onset and was less fatigued the day after completing therapy, although she continued to experience shortness of breath and muscle pain.

These cases along with our case represent anecdotal data, and are “hypothesis-generating,” which may provide clues as to what might be going on biologically.

There is a critical need to examine this hypothesis because a significant subset of immunocompromised patients with long COVID may benefit from antiviral treatment.

One might wonder why the acute COVID-19 in our patient did not progress to more critical disease despite the lack of any detectable antibody response to COVID -19 vaccine and to infection with SARS-CoV2. This question can be answered by the potential effect of cellular immunity that the patient acquired from the vaccine and from the natural infection. We think that the presence of partial effectiveness of cellular immunity was not able to get rid of the virus completely but effective enough to prevent progression to critical illness and death. This partial effectiveness of cellular immunity resulted in that the acute infection becoming persistent or chronic causing symptoms suggestive of long COVID.

One might also wonder why we used multiple antiviral medications to treat our patient. The decision to treat with several antiviral medications was based on the judgement that the patient is immunocompromised, and one antiviral might not be effective alone in eliminating the virus. In addition, there was a concern about the rebound phenomenon that has been reported with treatment with Paxlovid®. The evidence leaves most physicians favoring the idea that Paxlovid® knocks the virus down but doesn't knock it out completely.

Because our Immunocompromised patients didn't mount any detectable antibody response to COVID-19 vaccine and to the natural infection, she will continue to be at high risk for breakthrough infections and perhaps persistent viral replication. For this reason, our patient received a single dose of intramuscular Evusheld® to reduce the risk of breakthrough SARS-CoV-2 infection. Evusheld®, an injection of two different types of laboratory-made monoclonal antibodies (Cilgavimab and Tixageviman) with half-life of 90 days, was granted an emergency use authorization by the FDA for pre-exposure prophylaxis for immunocompromised people whom the COVID-19 vaccines are not effective, including solid organ transplant recipients, patients undergoing chemotherapy and people who take immunosuppressing medication.

Prophylactic treatment with Evusheld® in immunocompromised individuals reduced symptomatic SARS-CoV2 infection by 76.7% after 90 days according to the findings of the PROVENT study [28].

## 4 Conclusion

This is a case of long COVID in a lymphoma patient treated previously with Rituximab, who lacked antibody response to COVID-19 vaccine and to SARS-CoV-2 natural infection. Patient was treated successfully with COVID antiviral medications with complete resolution of her symptoms. Our case supports the hypothesis that immunocompromised patients who experienced long COVID symptoms may have persistent viral replication, and aggressive treatment with antiviral medications should be considered.

This case also strongly supports the hypothesis that newly diagnosed lymphoma patients should receive needed vaccines before starting treatment with rituximab or similar B-cell depleting drugs. It also supports the hypothesis that aggressive treatment with antivirals and monoclonal antibodies against COVID-19 is required in immunocompromised patients as early as possible to prevent complications among these vulnerable population.

## Author contribution statement

All authors listed have significantly contributed to the investigation, development and writing of this article.

## Funding

This research did not receive any specific grant from funding agencies in the public, commercial, or not-for-profit sectors.

## Informed consent

An informed consent was obtained from the patient prior to writing the manuscript.

## Data availability statement

The data that support the findings of this study are available from the corresponding author upon reasonable request.

## Declaration of competing interest

The authors declare that they have no known competing financial interests or personal relationships that could have appeared to influence the work reported in this paper
